# Preclinical Pharmacokinetic Studies of a Novel Diuretic Inhibiting Urea Transporters

**DOI:** 10.3390/molecules27082451

**Published:** 2022-04-11

**Authors:** Yue Xu, Hang Zhang, Nannan Li, Wen Ma, Shuyuan Wang, Jianguo Sun, Baoxue Yang

**Affiliations:** 1 State Key Laboratory of Natural and Biomimetic Drugs, Department of Pharmacology, School of Basic Medical Sciences, Peking University, Beijing 100191, China; yue_xu@bjmu.edu.cn (Y.X.); hangzhang@bjmu.edu.cn (H.Z.); 1610305107@pku.edu.cn (N.L.); 1911110073@bjmu.edu.cn (S.W.); 2 State Key Laboratory of Natural and Biomimetic Drugs, School of Pharmaceutical Sciences, Peking University, Beijing 100191, China; wen.ma@bjmu.edu.cn; 3 Key Lab of Drug Metabolism and Pharmacokinetics, State Key Laboratory of Natural Medicines, China Pharmaceutical University, 24 Tongjiaxiang Street, Nanjing 210009, China; jgsun@cpu.edu.cn

**Keywords:** LC–MS/MS, urea transporter, diuretics, tissue distribution, pharmacokinetics

## Abstract

Urea transporter (UT) inhibitors are a class of promising novel diuretics that do not cause the imbalance of Na^+^, K^+^, Cl^−^, and other electrolytes. In our previous studies, **25a**, a promising diuretic candidate inhibiting UT, was discovered and showed potent diuretic activities in rodents. Here, a sensitive liquid chromatography–tandem mass spectrometry method for the quantitation of **25a** in rat plasma, urine, feces, bile, and tissue homogenates was developed and validated to support the preclinical pharmacokinetic studies. The tissue distribution, excretion, and plasma protein binding were investigated in rats. After a single oral dose of **25a** at 25, 50, and 100 mg/kg, the drug exposure increased linearly with the dose. The drug accumulation was observed after multiple oral doses compared to a single dose. In the distribution study, **25a** exhibited a wide distribution to tissues with high blood perfusion, such as kidney, heart, lung, and spleen, and the lowest distribution in the brain and testis. The accumulative excretion rate of **25a** was 0.14%, 3.16%, and 0.018% in urine, feces, and bile, respectively. The plasma protein binding of **25a** was approximately 60% in rats and 40% in humans. This is the first study on the preclinical pharmacokinetic profiles of **25a**.

## 1. Introduction

Diuretics are the first-line drugs for hypertension, heart failure, and edema [1,2,3]. Classic diuretics (such as thiazide diuretics and loop diuretics) mainly have diuretic efficacy by interfering with the elimination of sodium, which could cause electrolyte imbalance and lead to several adverse reactions, such as metabolic alkalosis, hyponatremia, and hyperkalemia [4]. Sodium and urea both play essential roles in the urine concentration process in mammalian kidneys by maintaining the gradient of osmotic pressure from the cortex to the medulla of the kidney [5,6]. Urea, the metabolic end-product of amino acids, is mainly transported via urea transporters (UTs) [7]. UTs are specific transporters for urea, and the speed of urea transport is 10–100 times faster than passive diffusion crossing the cell membranes. UT-A (SLC14A2) and UT-B (SLC14A1) are the main isoforms of UTs, which play essential roles in the inner kidney urea cycles and manipulate the urine concentration in the kidney [8]. Several UT knockout animal models have demonstrated that UT-A and/or UT-B deficiency blocked inner kidney urea cycles and reduced urine concentrations, thus contributing to diuretic effects [9,10,11,12].

Recently, Yang et al. and Verkman et al. reported that several small-molecule inhibitors of UTs possess diuretic effects in mice and rats in a series of studies [13,14,15,16,17,18,19,20,21,22]. A novel diarylamide UT inhibitor **25a** with a potent inhibitory effect on UT-B at the sub-micromolar level (0.14 μM in rat and 0.48 μM in mice) was synthesized and investigated by our research group [23]. Additionally, **25a** exhibited a strong diuretic effect (three times higher urine output than vehicle control) on rats and mice orally administered without disturbing the Na^+^, K^+^, and Cl^−^ levels in the blood, and urea-selective diuresis continued for 8 h. Thus, it is necessary to further determine the drug-like properties of candidate **25a**. However, to the best of our knowledge, there is no report on the pharmacokinetic (PK) profiles (absorption, distribution, metabolism, and elimination (ADME)) of this kind of UT inhibitor.

The main aim of this study was (1) to develop and validate a sensitive and reliable liquid chromatography–tandem mass spectrometry (LC–MS/MS) method for quantitating **25a** in biomatrices according to the US FDA guidelines and (2) to elucidate the PK profiles, tissue distribution, and elimination in rats. We aimed to demonstrate the PK parameters in rats intravenously and orally administered **25a** at different dosages. The tissue distribution and elimination in rats were elucidated for further preclinical and clinical development of novel diuretics.

## 2. Results and Discussion

### 2.1. LC–MS/MS Method Development and Optimization

**Figure 1:**
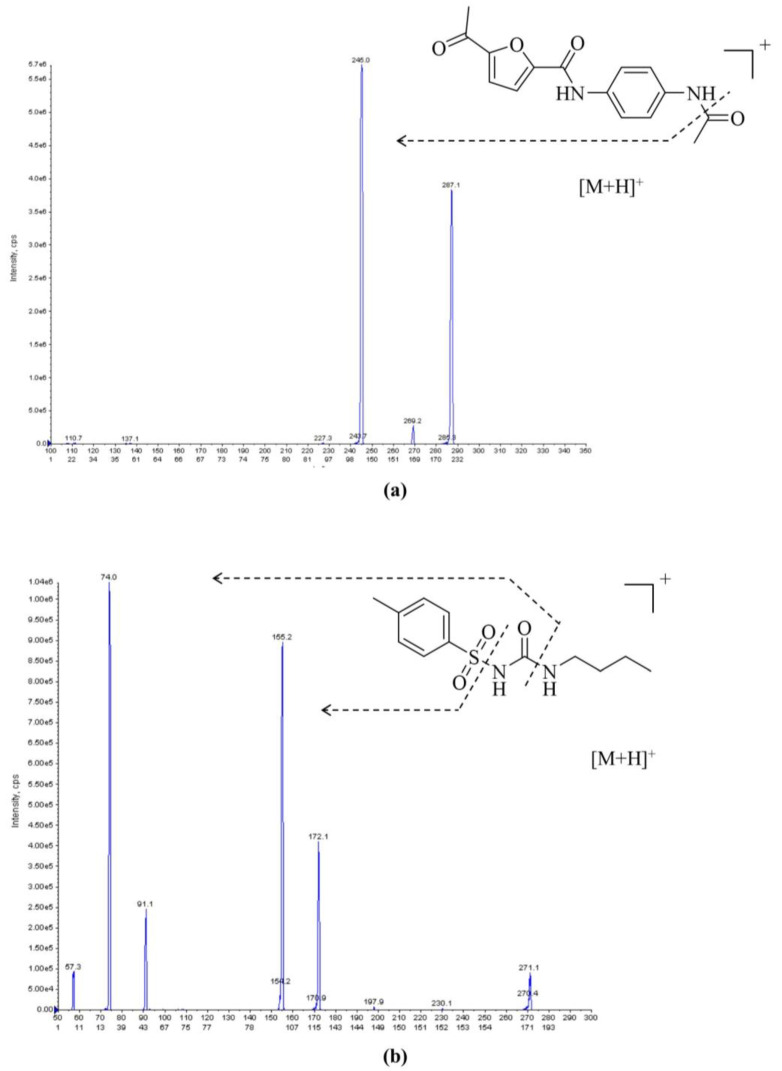
Representative MS/MS spectra of (**a**) **25a** and (**b**) IS in ESI positive-ion mode; the parent ions for **25a** and IS were 287.1 and 271.1, respectively.

First, for mass spectrometry (MS) method development, an electrospray ionization (ESI) source was tested in both positive-ion and negative-ion modes for **25a** and tolbutamide (internal standard, IS). The MS responses were much higher in the positive-ion mode. Hence, the adduct ions [M + H]^+^ were chosen as parent ions. The parent ions for **25a** and IS were 287.1 and 271.1, respectively. For product ion selection, the most abundant ion was 245.0 for **25a** and 74.0 for IS (Figure 1). The quantitation analysis of **25a** was conducted in multiple reaction monitoring (MRM) mode.

Second, for liquid chromatography (LC) analysis, a C18 column was used for reliability, compatibility, and reproductivity. Different mobile phase compositions were tested in different proportions to achieve better peak shape and retention. Methanol (MeOH), isopropanol (IPA), and acetonitrile (ACN) were selected as potential organic mobile phases for the assessment. Although IPA exhibited the strongest elution among the candidates, it led to increased baseline noise for the analyte, which confined the limits of quantitation (LOQ). Additionally, the high viscosity of IPA made the column pressure too high.

Lastly, ACN was employed as the organic phase to balance the elution ability and baseline noise. For the water phase, 0.1% (*v*/*v*) formic acid (FA) and 5 mM ammonium formate were added to improve chromatographic performance and achieve a proper retention time. A 5 min gradient elution process was employed to obtain high productivity for LC analysis (Table 1).

### 2.2. LC–MS/MS Validation

#### 2.2.1. Selectivity

**Figure 2:**
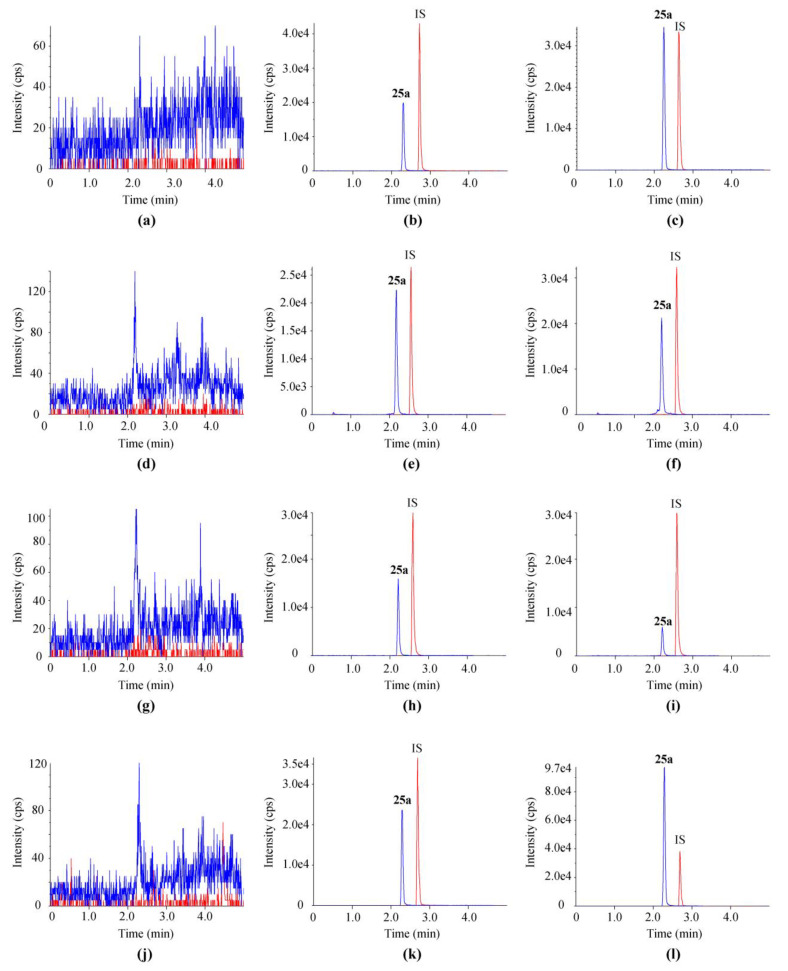
Typical chromatograms of **25a** and IS in (**a**,**d**,**g**,**j**) blank matrices, (**b**,**e**,**h**,**k**) blank matrices spiked with 500 ng/mL **25a**, and (**c**,**f**,**i**,**l**) sample 10 min after a single oral dose at 100 mg/kg. Matrices in (**a**–**c**) were plasma, in (**d**–**f**) were urine, in (**g**–**i**) were feces, and in (**j**–**l**) were bile.

Representative chromatograms of blank samples, blank matrices (plasma, urine, fecal homogenate, and bile of rats) spiked with **25a** and IS, and biological samples of rats post oral gavage are shown in Figure 2. All four kinds of matrices were taken from at least six individual rats to evaluate the selectivity of the LC–MS/MS method. No endogenous interference was observed at the retention time of **25a** (2.34 min) and IS (2.73 min).

#### 2.2.2. Linearity

The concentration range for the calibration curve of **25a** in four biological matrices was set as 1–1000 ng/mL. The linear regression was presented as *y* = *ax* + *b* with a 1/*x* weight factor. The correlation coefficients for all standard curves showed *r* > 0.99 (Table 2). The lower limit of quantitation (LLOQ) for **25a** determination was 1 ng/mL (signal-noise ratio > 10). The response of the LLOQ was over five times greater than that of the blank sample. The accuracy of LLOQ samples in all matrices was within ±20% (*n* = 6), and precision was within the acceptance criteria (<15%).

#### 2.2.3. Accuracy and Precision

Accuracy and precision were assessed by calculating the intraday and interday relative error (RE) and coefficient of variation (CV) of quality control (QC) samples at 3, 100, and 800 ng/mL (*n* = 6) on three successive days of analysis (Table 3). The intraday and interday RE for **25a** was −6.8%to 0.3%, −11.4% to 3.3%, 2.6% to 11.2%, and −12.1% to −7.8% in plasma, urine, feces, and bile, respectively. Both intraday and interday REs were within 8.0% in the four matrices for all QCs. These results demonstrated that the method for quantifying **25a** in rat matrices was precise and reliable.

#### 2.2.4. Recovery and Matrix Effect

The recovery and matrix effects of **25a** in plasma, urine, feces, and bile are shown in Table 4. The mean recovery of **25a** at the three concentration levels was 97.6% to 110.7%, 94.4% to 101.8%, 98.5% to 105.6%, and 88.7% to 94.1% in plasma, urine, feces, and bile, respectively, indicating that the extent of recovery was consistent and reproducible. The mean matrix effect was 103.7% to 107.5%, 98.0% to 99.3%, and 95.0% to 102.1% in plasma, urine, and feces, respectively, indicating a negligible matrix effect in rat biological matrices. It should be noted that the matrix effect in rat bile was 82.8% to 88.8%, with a CV less than 8.6%, showing a slight matrix suppression effect for the mass spectrum signal. The matrix effect may be due to endogenous components (such as bile acids/salts) interfering with the ionization of **25a** in the ion source [24,25].

#### 2.2.5. Stability Evaluation

The stability profiles of **25a** in the given matrices stored under different conditions (4 °C for 24 h, 25 °C for 4 h, three freeze–thaw cycles, and −80 °C for 30 days) are shown in Table 5. The RE (%) and CV (%) were within ±15% in the four matrices, demonstrating that the analytes were stable during sample preparation and storage.

#### 2.2.6. Dilution Integrity

In the dilution integrity assessment, 10,000 ng/mL **25a** was diluted with blank matrices to 500 ng/mL. As shown in Table S1 (see Supplementary Materials), the RE in rat plasma, urine, feces, and bile was 4.5%, 14.2%, −8%, and 0.4%, respectively, indicating the proper integrity and reliability of this dilution method (CV < 2.4%).

#### 2.2.7. Carry-Over

Carry-over between samples in the analytical method was evaluated by comparing the response of the double blank (DB) sample following an upper limit of quantitation (ULOQ) with that of the LLOQ. The mean carry-over of **25a** in the analysis ranged from 9.9% to 12.4%, while that of IS was less than 0.1% (Table S2), indicating the accuracy of the method in the determination of sample concentrations.

### 2.3. Pharmacokinetic and Pharmacodynamic Study

**Figure 3:**
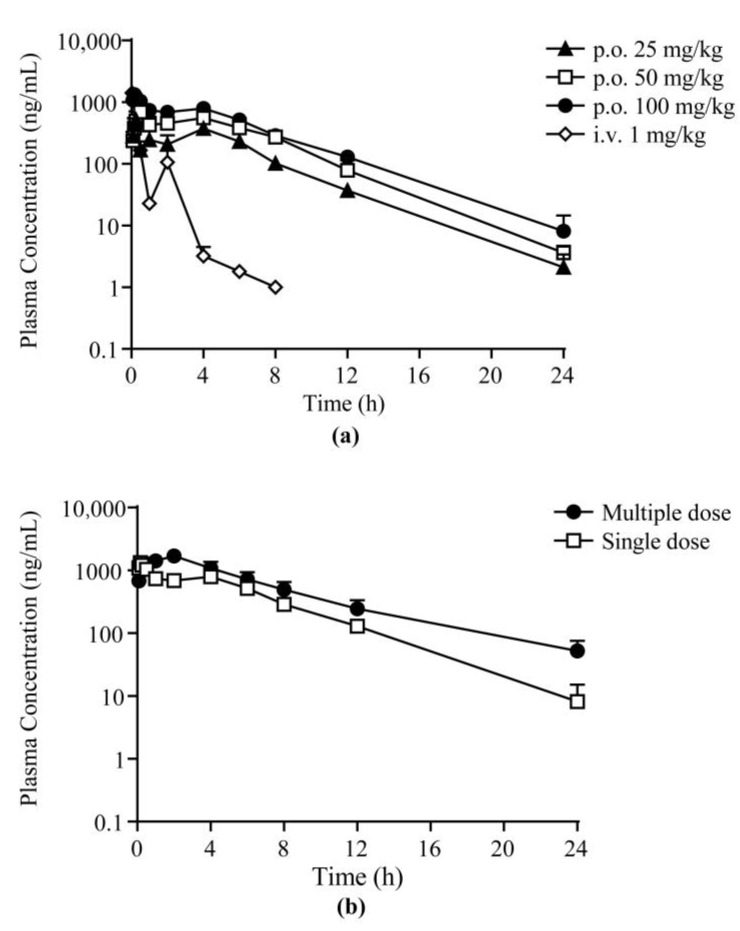
Mean plasma concentration–time profiles of **25a** in rats: (**a**) after a single p.o. and i.v. dose; (**b**) after multiple doses and single dose at 100 mg/kg body weight (mean ± SD, *n* = 6).

**Figure 4:**
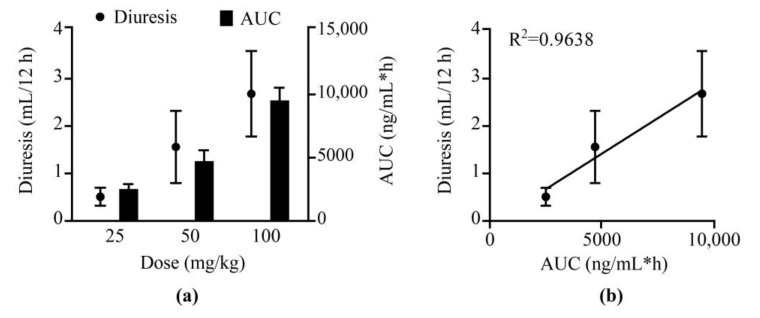
PK–PD profiles of **25a** after oral doses at 25, 50, and 100 mg/kg. (**a**) The relationship of dose with diuresis and AUC. (**b**) The relationship between AUC and diuresis in SD rats (mean ± SD, *n* = 6).

The validated LC–MS/MS method was employed in the PK study of **25a** in Sprague-Dawley (SD) rats after a single intravenous (i.v.) administration at 1 mg/kg, single oral (p.o.) doses at 25, 50, and 100 mg/kg, and successive oral doses at 100 mg/kg body weight. The mean plasma concentration–time profile of the single-dose groups and the comparison of the single oral dose and multiple doses are exhibited in Figure 3 and Table S3. The PK parameters using noncompartmental analysis (NCA) are shown in Table 6. The concentration of **25a** in rat plasma reached the maximum concentration (C_max_) within 0.5 h after oral administration, indicating fast oral absorption in rats. The half-life of **25a** was 2.6–3.0 h in rats after single oral doses, and the mean residence time (MRT) was approximately 5.4 h. The bioavailability was 20.1% ± 0.8%, 18.9% ± 1.7%, and 13.8% ± 0.8% at low, moderate, and high doses, respectively. The slight decrease in bioavailability may be due to the limitation of the water solubility of **25a** and absorption saturation, which need further exploration. As shown in Figure 4a, **25a** exhibited dose-dependent drug exposure in terms of C_max_ and area under the curve (AUC). In the pharmacokinetic and pharmacodynamic (PK–PD) relationship study, the volume of total urine output increment (diuresis) exhibited a dose-dependent relationship (Figure 4). The urine output increment per 12 h was 0.5 ± 0.2 mL, 1.6 ± 0.8 mL, and 2.7 ± 0.9 mL in rats administered with 25, 50, and 100 mg/kg **25a**, respectively. The AUC and diuresis showed a good linear regression (*R^2^* = 0.9638, *p* < 0.05, *n* = 6), indicating a high correlation between drug exposure and pharmacodynamic effect.

When compared with lead compound **1H**, the bioavailability of **25a** was approximately fourfold that of **1H** (4.38% ± 1.30%) [17]. Approximately 2 h after intravenous injection and 4 h after oral dosing, the second plasma peak concentration came, which indicated enterohepatic circulation in rats. This result was consistent with the two peaks of urine 4–6 h after **25a** treatment in the pharmacodynamic experiments [23]. Enterohepatic circulation also contributed to the longer half-life of **25a** (3.04 ± 0.70 h) compared with **1H** (0.93 ± 0.09 h) at the same dose [17]. Additionally, the mean C_max_ (1324.0 ± 227.5 ng/mL) of **25a** was also significantly higher than that of **1H** (75.4 ± 34.8 ng/mL), and a similar sharp increase was also found in the AUC (6921.7 ± 377.4 μg/L·h vs. 82.5 ± 40.0 μg/L·h). These data demonstrated successful chemical structure modifications for the diarylamide UT inhibitor **1H**.

After successive oral administration of **25a** twice per day for 7 days, the PK parameters significantly differed from those of the single-dose group. The plasma drug concentrations and AUC were increased significantly and were accompanied by prolonged t_1/2_ and decreased apparent clearance (CL/F), indicating the accumulation of **25a** in rats after multiple doses. Interestingly, the T_max_ was also prolonged from approximately 0.17 h to 2.0 h in the multiple-dose group. The accumulation may be due to absorption saturation and elimination [26].

In summary, compound **25a** showed a linear PK profile and PD–exposure relationship after single doses. The drug exposure of **25a** was in good line with the diuresis effects according to previous in vivo pharmacology studies in rats, demonstrating the high druggability of this novel diuretic [23]. Considering that our study was performed in rats, more studies should be conducted in nonrodent animals (e.g., beagle dogs) to confirm the PK profiles of these novel diuretics in the future.

### 2.4. Tissue Distribution

**Figure 5:**
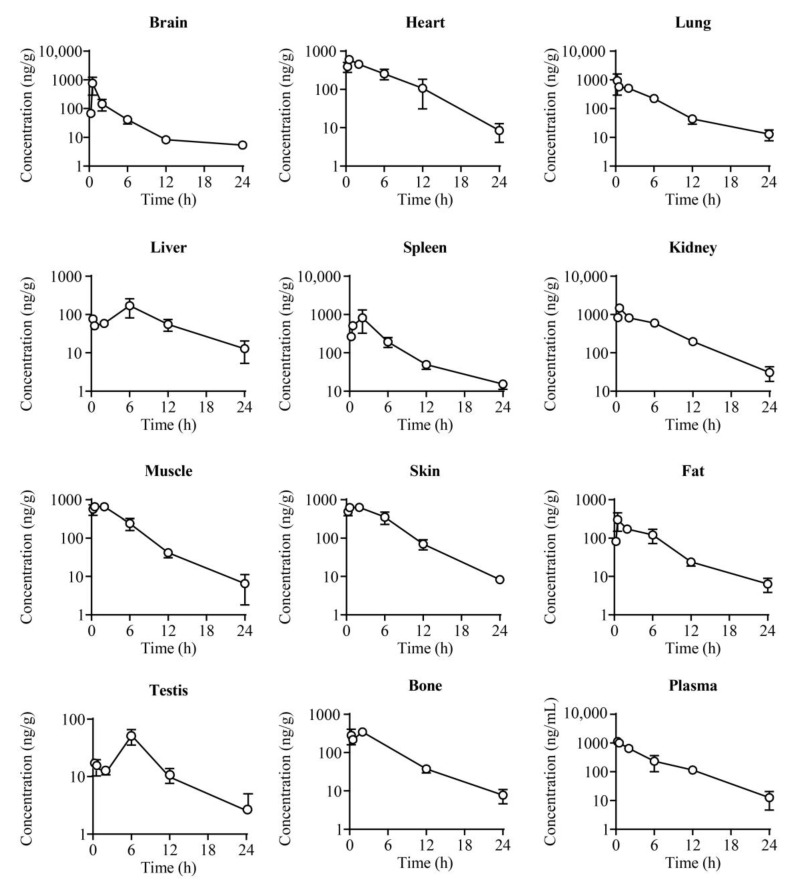
The concentration–time profiles of **25a** in plasma and different tissues of rats after a single oral dose of **25a** at 100 mg/kg (mean ± SD, *n* = 4).

The tissue concentration–time plots of **25a** at six timepoints (0.25, 0.5, 2, 6, 12, and 24 h) after a 100 mg/kg oral dose are exhibited in Figure 5 and Table S4. Compound **25a** was extensively distributed in most tissues of rats, and the drug concentration was high in organs with abundant hemoperfusions, such as the kidney, heart, lung, and spleen, among which the drug concentration was the highest in the kidney, with a tissue plasma distribution coefficient Kp = 1.67. The high kidney distribution is a good characteristic for diuretics targeting UTs, because the UTs in the renal inner medullary collecting duct (UT-A1/3) and renal vasa recta microvessels (UT-B) play vital roles in urea recycling and urine concentration [27,28]. The C_max_ and AUC showed a relatively lower drug distribution in the brain (Kp = 0.27) and testis (Kp = 0.06), indicating that **25a** was less permeable to the blood–brain barrier (BBB) and blood–testis barrier (BTB). Thus, the potential risk of **25a** for the central nervous system and male reproductive system is probably low.

### 2.5. Excretion Experiments

**Figure 6:**
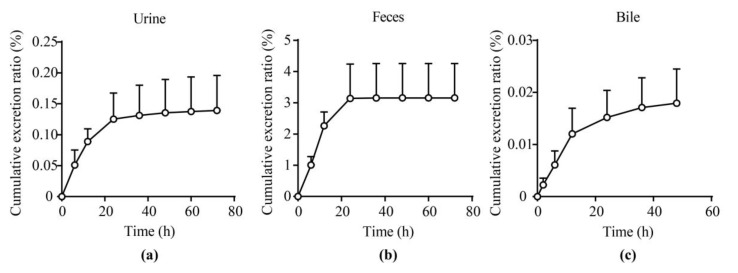
Excretion profiles of **25a** in (**a**) urine, (**b**) feces, and (**c**) bile of rats after a single oral administration at 100 mg/kg (mean ± SD, *n* = 4).

The cumulative excretion ratios of unchanged **25a** in the urine, feces, and bile of rats after a single oral dose at 100 mg/kg were 0.14% ± 0.05%, 3.16% ± 0.96%, and 0.018% ± 0.006%, respectively (Figure 6 and Tables S5–S7). The total cumulative excretion fractions of unchanged **25a** were less than 5% in urine, feces, and bile, indicating that **25a** may be mainly eliminated via metabolites. Further phenotyping and metabolite identification studies should be conducted to elucidate the major elimination forms of **25a** in the future.

### 2.6. Plasma Protein Binding and Blood Plasma Ratio

The plasma protein binding (PPB) and blood plasma ratio (BPR) are crucial parameters influencing the drug distribution and elimination in plasma [29]. The PPB profile of **25a** was evaluated by equilibrium dialysis, a classic method to assess the interactions of small molecules with proteins. The PPB of **25a** was tested at 50, 500, and 2500 ng/mL (Table 7). The mean PPB of **25a** in human and rat plasma was 38.8%–44.1% and 61.4%–64.5%, respectively, indicating a slight species difference, while the PPB of **25a** in both plasma matrices was relatively low to moderate compared with our reference compound warfarin (PPB = 98.5%). Additionally, the PPB at each concentration level showed no significant difference (*p* > 0.05, Student’s *t*-test, *n* = 3), indicating that **25a** bound with plasma protein in a concentration-independent manner. When considering the allometric scaling of PK parameters, the species difference of protein binding between humans and rats should be considered.

The BPR of **25a** in rats and humans was assessed. As shown in Table 8, the mean BPR of **25a** was 0.74–0.84 in rats and 1.22–1.39 in humans in a concentration-independent manner. In addition, a significant species difference between rats and humans was observed (*p* < 0.05, Student’s *t*-test, *n* = 3). These results suggested that **25a** can distribute to blood cells, and species differences should be considered in the allometric scaling.

## 3. Materials and Methods

### 3.1. Chemicals and Materials

The analyte **25a** was synthesized in our lab with purity > 99%. The internal standard (IS), tolbutamide, was purchased from Sigma–Aldrich (St. Louis, MO, USA). MeOH, IPA, ACN, ammonium formate, and FA were all MS-grade and purchased from Fisher (Fair Lawn, NY, USA). Sodium carboxymethylcellulose (CMC-Na), dimethyl sulfoxide (DMSO), and other reagents were analytically pure and purchased from Sigma-Aldrich. Distilled water was purchased from Watsons (Hong Kong, China).

### 3.2. LC–MS/MS Instrument Conditions

A UPLC BEH C18 column (2.1 mm × 50 mm, 1.7 μm, Waters, Milford, MA, USA) was employed for the chromatographic separation of **25a** and IS in a Dionex UltiMate 3000 Ultra–HPLC system (Thermo, San Jose, CA, USA). Gradient elution was employed with various proportions of 5 mM ammonium formate aqueous solution with 0.1% (*v*/*v*) FA and pure ACN at 0.3 mL/min (Table 1). The column temperature was 25 °C.

An API 4000 QTRAP mass spectrometer (AB SCIEX, Foster City, CA, USA) was employed in the mass spectrometric analysis. The type of ion source was electrospray ionization (ESI). Multiple–reaction monitoring (MRM) in positive–ion mode was employed for the quantitation analysis. The protonated molecular ion [M + H]^+^ for the analyte (**25a**) and IS was 287.0 and 271.1, respectively. The fragments of product ions are shown in Figure 1. The quantitative product ions for **25a** and IS were 245.0 and 74.0, respectively. The optimized source conditions were as follows: curtain gas, 10 psi; collision gas, 4 psi; ion spray voltage, 5000 V; source temperature, 600 °C; ion source gas 1, 55 psi; ion source gas 2, 60 psi. For **25a,** the declustering potential (DP) and collision energy (CE) were 65 V and 20 V, while those for IS were 60 V and 28 V, respectively. Analyst 1.6.1 software (AB SCIEX) was employed in data acquisition and analysis.

### 3.3. Sample Preparation

The analyte **25a** and IS were dissolved in DMSO to generate the stock solutions. The working calibration of **25a** was gradient-diluted with MeOH/H_2_O (1:1, *v*/*v*). The stop solution was 50 ng/mL of IS in ACN. Five microliters of working solutions were spiked with 45 μL of the blank biological matrices (rat plasma, urine, fecal homogenate, bile, and tissue homogenate) to obtain the calibration standards. The final concentrations of the standard samples were 1, 2, 10, 50, 250, 500, and 1000 ng/mL. The quality controls at three concentrations (3, 100, and 800 ng/mL) were generated similarly.

The protein precipitation method was employed for sample preparation. Briefly, 750 μL of stop solution was added to 50 μL of calibration standards and rat biological samples and vortexed for 1 min for protein precipitation. The mixture was centrifuged at 18,000× *g* for 10 min at 4 °C, and 2 μL of supernatant was injected for analysis.

### 3.4. Method Validation

The method was adequately validated in terms of the Guidance for Industry on Bioanalytical Method Validation (US FDA).

The selectivity was assessed in blank biological matrices, blank matrices spiked with working solutions, and biological samples of rats after p.o. administration of **25a**. Biological matrices from six individual rats were detected to distinguish the interference from the endogenous components in matrices and the LC–MS/MS systems.

A seven-point calibration curve (1, 2, 10, 50, 250, 500, and 1000 ng/mL) was fitted by plotting the **25a**/IS peak area ratios (*y*) versus the nominal concentrations (*x*) with a simple regression model combined with a weight factor of 1/*x*. The lower limit of quantitation (LLOQ) was the lowest concentration of **25a** in biological samples that could be determined accurately and that met S/N > 10 with a signal response at least five times higher than the blank samples. The RE and CV for LLOQ should be within ±20%. The peak areas of extracted QC samples (3, 100, and 800 ng/mL) were compared with post-extraction samples spiked with the analyte for recovery assessment. The matrix effect was assessed by comparing the peak area of samples containing matrix to that without matrix (*n* = 6).

The accuracy and precision were evaluated by calculating the intraday and interday RE and CV of the QC samples. The RE should be within ±15% of the theoretical concentrations (3, 100, and 800 ng/mL). The CV values should be no more than 15%. For the LLOQ, the cutoff values of RE and CV were 100% ± 20% and <20%. The stability of **25a** in the biological matrices (rat plasma, urine, fecal homogenate, and bile) was assessed at 3, 100, and 800 ng/mL (*n* = 6) under different storage conditions, which included storage at room temperature for 4 h, storage at 4 °C for 24 h, three freeze–thaw cycles, and storage at −80 °C for 30 days.

The carry-over effect was assessed by comparing the peak areas of a DB sample following ULOQ with that of LLOQ at the retention time of analytes. The acceptable criteria for **25a** and IS were RE ≤20% and 5%, respectively. The dilution integrity of the method was assessed by diluting 10,000 ng/mL **25a** to 500 ng/mL with the matrix. The RE should be within ±15% and the CV should be ≤15% (*n* = 6).

### 3.5. Pharmacokinetic Study in Rats

The animal experiments complied with the stipulations of the Ethics Committee of Peking University (Beijing, China). Male adult Sprague-Dawley rats (8 weeks old, 200 ± 20 g) were purchased from Vital River (Beijing, China) and fed freely at 22 ± 2 °C with 55% ± 5% humidity and 12 h/12 h light/dark cycles.

For oral administration groups, **25a** in 0.5% (*w*/*v*) CMC-Na saline solution was administered at 25, 50, and 100 mg/kg body weight. Blood samples were collected via orbital venous plexus into tubes with K_2_–EDTA on the ice at 0.083, 0.167, 0.25, 0.5, 1, 2, 4, 6, 8, 12, and 24 h. For the i.v. group, **25a** dissolved in a mixed solvent (DMSO, PEG400, and saline, 10:30:60, *v*/*v*/*v*) was intravenously injected at 1 mg/kg body weight via the tail vein. Blood was collected at 0.033, 0.083, 0.25, 0.5, 1, 2, 3, 4, 6, 12, and 24 h. Plasma was obtained by centrifugation at 3000 rpm for 10 min at 4 °C. For the PK–PD relationship study, the total urine of rats within 24 h was collected and weighed. The urine output was approximately calculated by assuming the density of urine was 1 g/mL. The diuresis effect was the urine output increment over the baseline urine output of each rat. The plasma and urine samples were stored at −80 °C until analysis. The PK parameters were calculated by DAS 3.2.8 software (Beijing, China) using the noncompartmental analysis (NCA) model. The oral bioavailability (F) was calculated by F = (AUC_p.o._ × Dose_i.v._)/(AUC_i.v._ × Dose_p.o._).

### 3.6. Tissue Distribution Study

Twenty-four SD rats were randomly divided into six groups and received a single oral dose of **25a** at 100 mg/kg. Blood and tissues samples (i.e., brain, heart, lungs, liver, spleen, kidneys, muscle, skin, fat, testes, and leg bones) were collected at 0.25 h, 0.5 h, 2 h, 6 h, 12 h, and 24 h after dosing. The tissue samples were rinsed with cold saline and dried on filter papers, weighed, and homogenized with fivefold volumes of ultrapure water using a homogenizer (T10, IKA, Staufen, Germany) on ice. The right shin bone of the rat was crushed and soaked in methanol for 12 h at 4 °C. Tissue homogenate or bone soak solution was prepared and analyzed using the above method. Samples were stored at −80 °C until analysis.

### 3.7. Excretion Experiment

Four SD rats were adapted in metabolic cages for 3 days in advance and then administrated with **25a** at 100 mg/kg orally. Feces and urine were collected and weighed at 0–6 h, 6–12 h, 12–24 g, 24–36 h, 36–48 h, 48–60 h, and 60–72 h after administration. Parallelly, four SD rats received bile duct cannulation and were given a single dose of **25a** at 100 mg/kg orally. Bile was collected and weighed at 0–2 h, 0–4 h, 0–6 h, 0–8 h, 0–10 h, 0–12 h, 0–24 h, 0–36 h, and 0–48 h after administration. All samples were stored at −80 °C until analysis.

### 3.8. Plasma Protein Binding Study

The plasma protein binding (PPB) ratio of **25a** was assessed via equilibrium dialysis in SD rats and human plasma. Four hundred microliters of plasma containing 50, 500, or 2500 ng/mL **25a** were loaded into the dialysis bags (plasma side), and the bags were placed into tubes containing 4 mL of the dialysis buffer (buffer side). The tubes were then rotated at approximately 100 rpm in a humidified incubator with 5% CO_2_ at 37 ± 1 °C for 5 h.

After the 5 h dialysis, aliquots of 50 μL samples were taken from both the buffer and the plasma sides. An equal volume of the opposite blank matrix (buffer or plasma) was added to each sample to reach a final volume of 100 μL with a volume ratio of plasma to dialysis buffer at 1:1 (*v*/*v*). All samples were further processed by adding 600 μL of stop solution (containing 10 ng/mL IS) to precipitate protein for LC–MS/MS analysis. The PPB ratio (%) was calculated by PPB% = 100% × (1 − Conc._buffer side_/Conc._plasma side_).

### 3.9. Blood Plasma Ratio Study

The blood plasma ratio of **25a** was investigated as previously reported [30]. Briefly, fresh whole blood was collected into collection tubes containing K_2_–EDTA as an anticoagulant from SD rats and healthy volunteers. Blood spiked with 50, 500, and 2500 ng/mL **25a** was incubated in the incubator with 5% CO_2_ at 37 ± 1 °C for 1 h. Some of the incubated samples were processed as blood samples; the remaining incubated samples were centrifuged to generate plasma samples. The concentration of **25a** in samples was determined using the validated LC–MS/MS method.

### 3.10. Statistical Analyses

Statistical analyses were performed with GraphPad Prism software (v7.0, GraphPad Software Inc., San Diego, CA, USA). An unpaired Student’s *t*-test was employed to determine statistical significance, α = 0.05.

## 4. Conclusions

This study is the first report on the preclinical pharmacokinetic profiles of **25a**, a promising novel diuretic agent targeting UTs. Firstly, we developed and validated a sensitive and reproducible LC–MS/MS method for the quantitation of **25a** in various rat biological matrices. Pharmacokinetic properties, including the absorption, distribution, metabolism, and excretion of **25a** in SD rats, were investigated systematically. After oral administration, **25a** exhibited a rapid absorption with a dose-dependent linear relationship between exposure (AUC or C_max_) and dose. The oral bioavailability was up to 20%, approximately four times higher than that of lead compound **1H** (4.4%), sharing a similar diarylamide scaffold. After multiple doses, drug accumulation was observed in rats. In addition, **25a** was distributed extensively to various tissues, such as the kidney, heart, lung, fat, and spleen, especially the target tissue, the kidney. After oral administration, the cumulative excretion fractions of **25a** were less than 5% in urine, feces, and bile, suggesting that **25a** was mainly eliminated via metabolism. The PPB of **25a** in rat and human plasma protein was relatively low. These preclinical PK studies can contribute to the further development of these novel diuretics targeting urea transporters.

## Figures and Tables

**Table 1:** LC elution gradient for separation of **25a** and IS.

Time (min)	Mobile Phase A (%)	Mobile Phase B (%)
0.0	95	5
0.5	30	70
1.0	0	100
3.0	0	100
3.1	95	5
5.0	95	5

Mobile phase A: 0.1% (*v*/*v*) FA and 5 mM ammonium formate in water; mobile phase B: acetonitrile.

**Table 2:** Linearity regression functions of **25a** in different matrices.

Matrix	*y* = *a × x* + *b* *		
	*a*	*b*	*r*
Plasma	0.01201	0.00391	0.9983
Urine	0.00965	0.00204	0.9997
Feces	0.00967	0.00218	0.9996
Bile	0.00708	0.00325	0.9989
Kidney	0.01200	0.00390	0.9995
Heart	0.01180	0.00314	0.9997
Lung	0.01090	0.00400	0.9996
Spleen	0.01060	0.00646	0.9997
Muscle	0.01190	0.00332	0.9998
Skin	0.01030	0.00640	0.9994
Bone	0.00926	0.00694	0.9997
Brain	0.01170	0.00395	0.9999
Fat	0.01100	0.00259	0.9997
Liver	0.00961	0.00599	0.9996
Testis	0.01000	0.00878	0.9997

* Linear regression with 1/*x* weight; *r*: correlation coefficient.

**Table 3:** LLOQ, accuracy, and precision of **25a** in rat plasma, urine, feces, and bile (*n* = 6).

Matrix	Sample	Nominal Conc. (ng/mL)	Intraday		Interday	
			RE (%)	CV (%)	RE (%)	CV (%)
Plasma	LLOQ	1	1.2	6.0	−4.6	8.4
	LQC	3	−3.9	2.1	−6.8	5.1
	MQC	100	0.3	1.7	−2.8	5.9
	HQC	800	−3.0	1.6	−3.4	3.8
Urine	LLOQ	1	−14.9	5.8	−15.2	0.4
	LQC	3	−11.4	8.0	−10.6	2.1
	MQC	100	3.3	1.7	−0.7	6.3
	HQC	800	−3.0	4.3	−4.7	3.0
Feces	LLOQ	1	7.0	2.3	9.1	2.4
	LQC	3	2.6	5.4	7.4	3.9
	MQC	100	11.2	4.1	7.5	3.2
	HQC	800	3.9	4.2	4.5	1.2
Bile	LLOQ	1	15.2	2.9	7.9	11.2
	LQC	3	−12.1	4.2	−9.7	3.5
	MQC	100	−7.8	2.8	−8.0	0.6
	HQC	800	−11.2	3.5	−11.6	0.8

**Table 4:** Recovery and matrix effect of **25a** in plasma, urine, feces, and bile (*n* = 6).

Matrix	Quality Control	Nominal Conc. (ng/mL)	Recovery			Matrix Effect		
			Mean (%)	SD (%)	CV (%)	Mean (%)	SD (%)	CV (%)
Plasma	LQC	3	97.6	6.0	6.1	105.4	2.8	2.5
	MQC	100	98.2	4.5	4.5	107.5	2.1	2.1
	HQC	800	110.7	4.7	4.7	103.7	4.2	4.2
Urine	LQC	3	101.8	7.6	7.4	98.2	5.3	4.8
	MQC	100	94.4	8.4	8.9	99.3	2.6	2.4
	HQC	800	100.9	3.6	3.5	98.0	0.9	0.8
Feces	LQC	3	98.5	8.0	7.5	102.1	8.0	6.8
	MQC	100	105.6	2.8	5.4	95.0	2.4	5.0
	HQC	800	103.8	10.8	9.4	97.1	9.6	7.9
Bile	LQC	3	94.1	10.0	10.6	88.8	9.0	8.1
	MQC	100	88.7	3.1	3.5	82.8	9.6	8.6
	HQC	800	92.5	10.3	11.1	83.5	5.5	5.0

**Table 5:** Stability study of **25a** in rat plasma, urine, feces, and bile under different conditions (*n* = 6).

Matrix	Quality Control	Nominal Conc. (ng/mL)	25 °C for 4 h		4 °C for 24 h		Three Freeze–Thaw Cycles		−80 °C for 30 days	
			RE (%)	CV (%)	RE (%)	CV (%)	RE (%)	CV (%)	RE (%)	CV (%)
Plasma	LQC	3	−4.9	2.0	2.8	5.6	−3.6	1.4	1.1	5.6
	MQC	100	−2.5	2.2	3.7	1.3	−4.0	2.1	3.7	1.3
	HQC	800	−3.1	0.5	−4.5	1.7	−7.6	2.1	−4.5	1.7
Urine	LQC	3	3.2	6.7	−8.3	3.1	5.8	10.7	6.2	3.1
	MQC	100	4.3	1.7	2.5	1.7	−5.2	4.2	2.5	1.7
	HQC	800	−2.3	4.4	−2.3	3.9	−1.5	3.5	−2.3	3.9
Feces	LQC	3	−5.2	4.9	3.9	7.4	−5.2	2.9	1.2	7.4
	MQC	100	1.3	3.4	4.8	3.2	−3.2	3.1	4.8	3.2
	HQC	800	−2.4	0.9	−5.3	1.5	−9.1	4.9	−5.3	1.5
Bile	LQC	3	−14.9	4.7	−14.7	5.8	−5.8	10.7	−2.1	5.8
	MQC	100	−5.1	9.0	−3.9	2.2	−5.2	4.2	−3.9	2.2
	HQC	800	−7.8	3.7	−7.1	3.5	−12.2	3.5	−7.1	3.5

**Table 6:** Pharmacokinetic parameters of **25a** in rats with single intravenous injection, oral administration, and multiple oral doses. Data are presented as the mean ± SD (*n* = 6).

Parameter	Unit	Single i.v. 1 mg/kg	Single p.o. 25 mg/kg	Single p.o. 50 mg/kg	Single p.o. 100 mg/kg	Multiple p.o. 100 mg/kg
*t*_1/2_	h	0.89 ± 0.52	2.86 ± 0.29	2.63 ± 0.20	3.04 ± 0.70	5.03 ± 0.82
*t*_max_	h	-	0.25 ± 0.00	0.50 ± 0.00	0.17 ± 0.04	2.00 ± 0.00
*C*_max_^1^	ng/mL	1711.0 ± 397.7	515.0 ± 106.7	693.2 ± 260.4	1324.0 ± 227.5	1711.7 ± 185.0
AUC_0–t_	μg/L·h	500.9 ± 124.3	2517.9 ± 395.2	4729.6 ± 854.3	6921.7 ± 377.4	11,753.9 ± 1145.6
AUC_0–∞_	μg/L·h	502.7 ± 124.4	2526.5 ± 362.4	4743.3 ± 854.4	6957.4 ± 345.1	12,132.7 ± 1187.2
MRT	h	0.6 ± 0.1	5.2 ± 0.4	5.4 ± 0.8	5.3 ± 0.3	6.5 ± 0.6
V/F ^2^	L/kg	2.6 ± 1.4	40.9 ± 7.4	40.1 ± 10.8	63.0 ± 17.6	59.8 ± 11.8
CL/F ^3^	L/kg/h	2.1 ± 0.6	9.9 ± 1.6	10.5 ± 2.2	14.4 ± 0.7	8.2 ± 0.8
F	%	-	20.1 ± 0.8	18.9 ± 1.7	13.8 ± 0.8	23.5 ± 2.3

^1^ C_0_, ^2^ V and ^3^ CL for i.v. group.

**Table 7:** Protein binding of **25a** in human and rat plasma. Data are presented as the mean ± SD (*n* = 3).

Species	Plasma Protein Binding (%)		
	50 ng/mL	500 ng/mL	2500 ng/mL
Human	44.1 ± 2.5	38.8 ± 12.0	40.5 ± 2.5
Rat	63.2 ± 3.3	61.4 ± 1.2	64.5 ± 2.5

**Table 8:** Blood plasma ratio of **25a** in humans and rats. Data are presented as the mean ± SD (*n* = 3).

Species	Blood Plasma Ratio		
	50 ng/mL	500 ng/mL	2500 ng/mL
Human	1.39 ± 0.14	1.28 ± 0.05	1.22 ± 0.32
Rat	0.84 ± 0.26	0.74 ± 0.06	0.79 ± 0.15

## Data Availability

Data is contained within the article or Supplementary Materials.

## Supplementary Materials

The following supporting information can be downloaded at https://www.mdpi.com/article/10.3390/molecules27082451/s1: Figure S1. Evaluation of carry-over effect. Representative chromatograms of **25a** and IS in (a) ULOQ sample (1000 ng/mL) in rat plasma; (b) double blank sample following the ULOQ; Table S1. Dilution integrity test for **25a** in rat plasma, urine, feces, and bile. Data are presented as the mean ± SD (*n* = 6); Table S2. The carry-over test for **25a** in rat plasma, urine, feces, and bile. Data are presented as the mean ± SD (*n* = 6); Table S3. Mean plasma concentration data of **25a** in rats after a single oral dose at 25, 50, and 100 mg/kg and multiple doses at 100 mg/kg. Data are presented as the mean ± SD (ng/mL, *n* = 6); Table S4. Tissue distribution parameters of **25a** in rats after a single oral dose at 100 mg/kg. Data are presented as the mean ± SD (*n* = 4); Table S5. Urine excretion profile of **25a** in rats a single oral dose 100 mg/kg. Data are presented as the mean ± SD (*n* = 4); Table S6. Fecal excretion profile of **25a** in rats after a single oral dose ay 100 mg/kg. Data are presented as the mean ± SD (*n* = 4); Table S7. Bile excretion profile of **25a** in rats after a single oral dose ay 100 mg/kg. Data are presented as the mean ± SD (*n* = 4).

## Sample Availability

Samples of Compound **25a** are available from the authors.
