# Supplementary material for: Preclinical Pharmacokinetic Studies of a Novel Diuretic Inhibiting Urea Transporters
Source: Molecules. 2022 Apr 11;27(8):2451. doi: 10.3390/molecules27082451 (PMC9027532; doi:10.3390/molecules27082451)
Supplement: Supplementary file 1 [file molecules-27-02451-s001.zip › molecules-1656630-supplementary.pdf]

Preclinical Pharmacokinetic Studies of a Novel Diuretic Inhibiting Urea Transporters

Yue Xu [1], Hang Zhang [1], Nannan Li [1], Wen Ma [2], Shuyuan Wang [1], Jianguo Sun [3] and Baoxue Yang [1,*]

[1] State Key Laboratory of Natural and Biomimetic Drugs, Department of Pharmacology, School of Basic Medical Sciences, Peking University, Beijing, 100191, China; yue_xu@bjmu.edu.cn (Y.X.); hangzhang@bjmu.edu.cn (H.Z.); 1610305107@pku.edu.cn (N.L.); 1911110073@bjmu.edu.cn (S.W.)

[2] State Key Laboratory of Natural and Biomimetic Drugs, School of Pharmaceutical Sciences, Peking University, Beijing, 100191, China; wen.ma@bjmu.edu.cn

[3] Key Lab of Drug Metabolism and Pharmacokinetics, State Key Laboratory of Natural Medicines, China Pharmaceutical University, 24 Tongjiaxiang Street, Nanjing, 210009, Jiangsu, China; jgsun@cpu.edu.cn

* Correspondence: baoxue@bjmu.edu.cn; Tel.: +86-10-8280-5559

Supplementary marteials

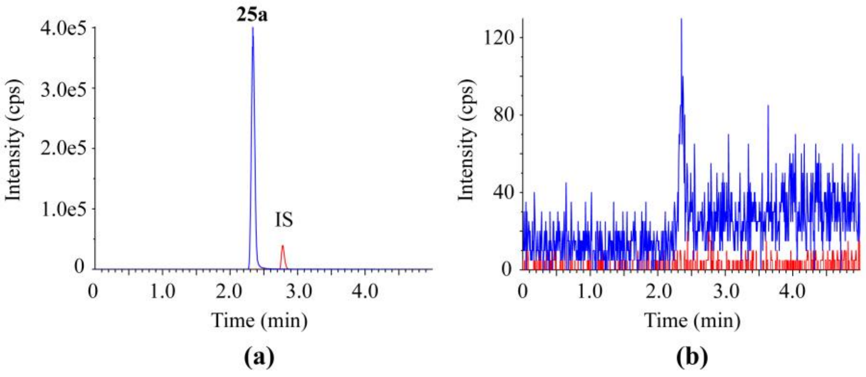

Figure S1. Evaluation of carry-over effect. Representative chromatograms of **25a** and IS in **(a)** ULOQ sample (1000 ng/mL) in rat plasma; **(b)** double blank sample following the ULOQ.

Table S1. Dilution integrity test for **25a** in rat plasma, urine, feces and bile. Data were presented as the mean ± SD (n = 6).

Matrix	Nominal Conc. (ng/mL)	Measured Conc. (ng/mL)	RE (%)	CV (%)
Plasma	10,000	10,450.0 ± 236.3	4.5	2.3
Urine	10,000	11,416.7 ± 219.2	14.2	1.9
Feces	10,000	9201.7 ± 144.8	-8.0	1.6
Bile	10,000	10,043.3 ± 241.0	0.4	2.4

Table S2. The carry-over test for **25a** in rat plasma, urine, feces and bile. Data were presented as the mean ± SD (n = 6).

Matrix	DB[1] Area (cps)	LLOQ Area (cps)	Carryover
Plasma	129 ± 16	1177 ± 131	11.0% ± 1.0%
Urine	236 ± 37	1903 ± 100	12.4% ± 2.3%
Feces	181 ± 63	1823 ± 186	9.9% ± 3.2%
Bile	215 ± 52	1752 ± 515	12.3% ± 3.0%

[1] DB: Double blank sample following a ULOQ sample

Table S3. Mean plasma concentration data of **25a** in rats after a single oral dose at 25, 50 and 100 mg/kg and multiple doses at 100 mg/kg. Data were presented as the mean ± SD (ng/mL, n = 6).

Time (h)	Single *p.o.* 25 mg/kg	Single *p.o.* 50 mg/kg	Single *p.o.* 100 mg/kg	Multiple *p.o.* 100 mg/kg
0.083	281.5 ± 62.3	236.3 ± 93.5	1078.4 ± 286.8	681.3 ± 97.0
0.167	430.7 ± 115.8	379.7 ± 173.4	1324.0 ± 234.6	935.6 ± 101.8
0.25	515.0 ± 122.5	495.7 ± 211.4	1211.0 ± 196.1	1089.5 ± 191.7
0.5	167.0 ± 33.5	693.2 ± 271.5	1034.4 ± 124.4	1264.0 ± 168.2
1	244.7 ± 92.5	428.8 ± 149.2	733.6 ± 134.2	1411.4 ± 282.9
2	206.2 ± 83.7	453.7 ± 97.6	687.4 ± 145.8	1711.7 ± 185.0
4	372.5 ± 74.3	553.3 ± 97.8	1129.2 ± 245.4	1075.2 ± 110.2
6	229.8 ± 49.7	382.8 ± 165.8	834.6 ± 178.5	717.4 ± 195.7
8	101.8 ± 7.7	271.0 ± 75.1	595.6 ± 126.4	493.7 ± 111.2
12	36.8 ± 2.7	78.3 ± 36.1	172.9 ± 36.7	244.8 ± 73.6
24	2.1 ± 1.3	3.6 ± 1.1	10.9 ± 6.6	28.1 ± 85.9

Table S4. Tissue distribution parameters of **25a** in rats after a single oral dose at 100 mg/kg. Data were presented as the mean ± SD (n = 4).

Tissue	$t_{1/2}$ (h)	T_{max} (h)	C_{max} (ng/mL)	AUC_{0-t} (ng/mL*h)	Kp -
Brain	6.1 ± 2.8	0.9 ± 0.6	819.5 ± 768.7	1393.4 ± 602.4	0.27
Heart	3.5 ± 0.6	0.8 ± 0.7	631.3 ± 64.8	4163.1 ± 1006.9	0.80
Lung	3.6 ± 0.7	0.4 ± 0.1	1128.8 ± 1005.5	3717.9 ± 880.5	0.72
Liver	6.3 ± 3.4	3.6 ± 2.5	183.3 ± 143.8	1648.3 ± 1000.1	0.22
Spleen	4.5 ± 0.8	0.9 ± 0.6	933.8 ± 792.8	4268.3 ± 2625.5	0.82
Kidney	4.2 ± 0.8	0.4 ± 0.1	1478.8 ± 472.9	8673.5 ± 1507.1	1.67
Muscle	3.2 ± 0.4	0.8 ± 0.7	727.5 ± 215.8	4000.0 ± 790.5	0.79
Skin	3.6 ± 0.5	2.6 ± 2.1	701.3 ± 123.5	4833.9 ± 1084.6	0.93
Fat	6.6 ± 4.2	1.3 ± 0.8	333.4 ± 238.5	1573.4 ± 650.2	0.31
Testis	NA	6.0 ± 0.0	50.6 ± 26.4	337.6 ± 143.9	0.06
Bone	3.7 ± 0.5	1.1 ± 0.9	412.8 ± 149.7	2680.0 ± 660.3	0.40
Plasma	5.7 ± 4.8	0.3 ± 0.0	1121.3 ± 131.9	5197.6 ± 1591.9	1.00

[1] NA: Not Available for no enough drug concentrations

Table S5. Urine excretion profile of **25a** in rats a single oral dose 100 mg/kg. Data were presented as the mean ± SD (n = 4).

Timepoint (h)	Urine (mL)	Concentration (ng/mL)	Accumulated Amount (mg)	The cumulative excretion ratio (%)
0-6	7.3 ± 3.0	1865.0 ± 576.4	0.0129 ± 0.0059	0.051 ± 0.021
6-12	6.5 ± 1.5	1696.3 ± 1,244.3	0.0221 ± 0.0046	0.089 ± 0.018
12-24	9.1 ± 2.2	869.3 ± 442.0	0.0305 ± 0.0066	0.125 ± 0.037
24-36	8.3 ± 2.6	132.4 ± 108.1	0.0317 ± 0.0077	0.131 ± 0.042
36-48	9.8 ± 2.7	104.4 ± 95.7	0.0327 ± 0.0085	0.135 ± 0.047
48-60	9.8 ± 2.8	49.6 ± 31.9	0.0332 ± 0.0089	0.137 ± 0.049
60-72	14.9 ± 6.4	32.5 ± 11.2	0.0337 ± 0.0090	0.139 ± 0.049

Table S6. Fecal excretion profile of **25a** in rats a single oral dose 100 mg/kg. Data were presented as the mean ± SD (n = 4).

Timepoint (h)	Feces (g)	Concentration (ng/mL)	Accumulated Amount (mg)	The cumulative excretion ratio (%)
0-6	7.7 ± 0.8	6547.5 ± 1407.8	0.248 ± 0.056	1.009 ± 0.233
6-12	7.1 ± 2.0	9237.5 ± 2337.8	0.565 ± 0.130	2.261 ± 0.383
12-24	7.1 ± 2.0	6053.3 ± 4545.2	0.798 ± 0.309	3.139 ± 0.954
24-36	3.6 ± 1.0	188.2 ± 121.9	0.802 ± 0.310	3.153 ± 0.957
36-48	9.8 ± 1.7	7.0 ± 8.8	0.802 ± 0.310	3.155 ± 0.956
48-60	4.7 ± 3.0	2.1 ± 3.1	0.802 ± 0.310	3.155 ± 0.956
60-72	13.0 ± 4.0	3.8 ± 3.8	0.802 ± 0.310	3.156 ± 0.955

Table S7. Bile excretion profile of **25a** in rats a single oral dose 100 mg/kg. Data were presented as the mean ± SD (n = 4).

Timpoint (h)	Bile (mL)	Concentration (ng/mL)	Accumulated Amount (μg)	The cumulative excretion ratio (%)
0-2	2.4 ± 0.9	220.5 ± 44.4	0.531 ± 0.233	0.0022 ± 0.0011
0-4	3.2 ± 0.6	192.3 ± 52.3	1.138 ± 0.434	0.0048 ± 0.0022
0-6	1.8 ± 0.5	161.3 ± 55.4	1.451 ± 0.481	0.0061 ± 0.0023
0-8	1.9 ± 0.5	169.5 ± 57.2	1.783 ± 0.489	0.0074 ± 0.0023
0-10	2.7 ± 0.9	156.8 ± 23.8	2.215 ± 0.603	0.0092 ± 0.0026
0-12	4.7 ± 3.0	147.3 ± 27.7	2.888 ± 0.929	0.0120 ± 0.0043
0-24	9.9 ± 3.6	83.5 ± 19.9	3.664 ± 1.010	0.0152 ± 0.0045
0-36	12.3 ± 3.2	40.7 ± 21.1	4.113 ± 1.058	0.0171 ± 0.0049
0-48	9.0 ± 2.5	17.9 ± 17.1	4.300 ± 1.184	0.0179 ± 0.0057
